# Development and Validation of Reverse Transcription Loop-Mediated Isothermal Amplification (RT-LAMP) for Rapid Detection of ZIKV in Mosquito Samples from Brazil

**DOI:** 10.1038/s41598-019-40960-5

**Published:** 2019-03-14

**Authors:** Severino Jefferson Ribeiro da Silva, Marcelo Henrique Santos Paiva, Duschinka Ribeiro Duarte Guedes, Larissa Krokovsky, Fábio Lopes de Melo, Maria Almerice Lopes da Silva, Adalúcia da Silva, Constância Flávia Junqueira Ayres, Lindomar J. Pena

**Affiliations:** 1 Department of Virology, Oswaldo Cruz Foundation (Fiocruz), Recife, Pernambuco Brazil; 2 Agreste Academic Center, Federal University of Pernambuco (UFPE), Caruaru, Pernambuco Brazil; 3 Department of Entomology, Oswaldo Cruz Foundation (Fiocruz), Recife, Pernambuco Brazil; 4 Department of Parasitology, Oswaldo Cruz Foundation (Fiocruz), Recife, Pernambuco Brazil

## Abstract

The rapid spread of Zika virus (ZIKV) represents a global public health problem, especially in areas that harbor several mosquito species responsible for virus transmission, such as Brazil. In these areas, improvement in mosquito control needs to be a top priority, but mosquito viral surveillance occurs inefficiently in ZIKV-endemic countries. Quantitative reverse transcription PCR (qRT-PCR) is the gold standard for molecular diagnostic of ZIKV in both human and mosquito samples. However, the technique presents high cost and limitations for Point-of-care (POC) diagnostics, which hampers its application for a large number of samples in entomological surveillance programs. Here, we developed and validated a one-step reverse transcription LAMP (RT-LAMP) platform for detection of ZIKV in mosquito samples. The RT-LAMP assay was highly specific for ZIKV and up to 10,000 times more sensitive than qRT-PCR. Assay validation was performed using 60 samples from *Aedes aegypti* and *Culex quinquefasciatus* mosquitoes collected in Pernambuco State, Brazil, which is at the epicenter of the Zika epidemic. The RT-LAMP had a sensitivity of 100%, specificity of 91.18%, and overall accuracy of 95.24%. Thus, our POC diagnostics is a powerful and inexpensive tool to monitor ZIKV in mosquito populations and will allow developing countries to establish better control strategies for this devastating pathogen.

## Introduction

Zika virus (ZIKV) is a mosquito-borne flavivirus, first identified in a rhesus monkey from Uganda in 1947 and isolated from *Aedes africanus* mosquitoes in 1948^1^. For nearly 60 years few ZIKV cases in human have been reported. However, in 2007 a large ZIKV outbreak occurred in the Yap Island, Federated States of Micronesia. In 2013, the virus was detected in French Polynesia and rapidly spread throughout the Pacific^2,3^. In these outbreaks, most ZIKV infections have been asymptomatic and, when present, symptoms include rash, fever, headache, and arthralgia^4^. However, the unprecedented epidemics of developmental defects first reported in 2015 in newborns from Brazil and neurological complications associated with the infection such as Guillain-Barré syndrome (GBS) mobilized public health officials and scientists around the world to fill knowledge gaps of this until then overlooked pathogen^5–7^.

ZIKV is an arbovirus member of the genus *Flavivirus* in the family *Flaviviridae*. The ZIKV genome consists of a single positive-sense single-stranded RNA (+ssRNA), with approximately 11 Kb in length. Other important viruses within this genus include yellow fever virus (YFV), dengue 1–4 virus (DENV 1–4), Japanese encephalitis virus (JEV) and West Nile virus (WNV)^8^.

Mosquitoes from the genus *Aedes* are widespread in tropical and subtropical regions of the world and have been postulated as the main vector for ZIKV^9^. However, different studies have suggested that the southern house mosquito *Culex quinquefasciatus* mosquitoes could act as another important ZIKV vector^10–13^. Moreover, many ZIKV strains have been isolated from *Anopheles*, *Mansonia*, *Culex* and *Aedes* mosquitoes^14^. Non-vector-borne transmission of ZIKV can occur through blood transfusion, transplacentally, perinatally and sexually^15^. Given the lack of vaccines and antivirals against ZIKV, vector control remains the most effective manner to limit virus spread and the size of outbreaks^16^.

ZIKV surveillance in insect vectors is an important tool for identifying viral circulation and potential entry points, therefore contributing to prevent outbreaks of disease^17^. This virus has spread rapidly particularly in developing countries that lacks good sanitation infrastructure and harbors several mosquito species competent for ZIKV transmission. In these areas, improvement in mosquito control needs to be a top priority, but occurs inefficiently in ZIKV-endemic countries, such as Brazil^17–19^. Surveillance of ZIKV in mosquitoes sheds lights into virus dynamics and allows early detection of new introductions before the virus become widespread in vector and host populations. In addition, surveillance data allows the evaluation of trends and the impact of vector control programs^20^.

Currently, quantitative reverse transcription PCR (qRT-PCR) is the gold standard for molecular diagnostic of ZIKV in both humans and mosquito samples^21,22^. However, qRT-PCR is expensive, requires highly specialized manpower, and involves costly and sophisticated equipment for amplification and detection of the viral genome. These drawbacks make the technique unsuitable for large-scale applications in low-resource settings areas, which negatively impact the establishment of effective disease control programs^23,24^.

Point-of-care (POC) molecular diagnostic platforms may address these concerns and increase the diagnostic capacity of ZIKV-affected countries. RT-LAMP is a promising tool that allows rapid, simple and practical diagnosis of a number of pathogens^25–27^. Considering the advantages of rapid amplification, simple operation, low cost, high sensitivity and specificity, RT-LAMP has potential applications for clinical diagnosis as well as for surveillance of infectious diseases in developing countries^28^. Differently from the qRT-PCR assay, detection of RT-LAMP amplification products can achieved by naked eye analysis through color change of the reaction tube^29^. For this purpose, different LAMP assays have been developed for detecting the ZIKV since its emergence in the Western hemisphere^30–38^. However, most ZIKV LAMP assays developed to date evaluated only handful mosquito samples, which raise concerns about their fitness for ZIKV detection in the field. Moreover, many of the developed ZIKV LAMP assays still require special equipments for virus detection, which limits its applicability in low-resource scenarios.

In the present study, we developed and validated a one-step reverse transcription LAMP (RT-LAMP) platform for detection of ZIKV in both laboratory and wild-caught mosquitoes. The RT-LAMP assay described here enables the diagnosis of ZIKV in mosquito samples as fast as 20 minutes even in the absence of RNA isolation from the samples. In addition, it does not require highly trained workforce and does not involve expensive and sophisticated equipment for amplification and virus detection. Our point-of-care test is a powerful and inexpensive tool to monitor ZIKV in mosquito populations and will allow developing countries to establish better and timely decisions regarding ZIKV control strategies.

## Results

### Detection of ZIKV in *Aedes aegypti* under controlled conditions

**Figure 1:**
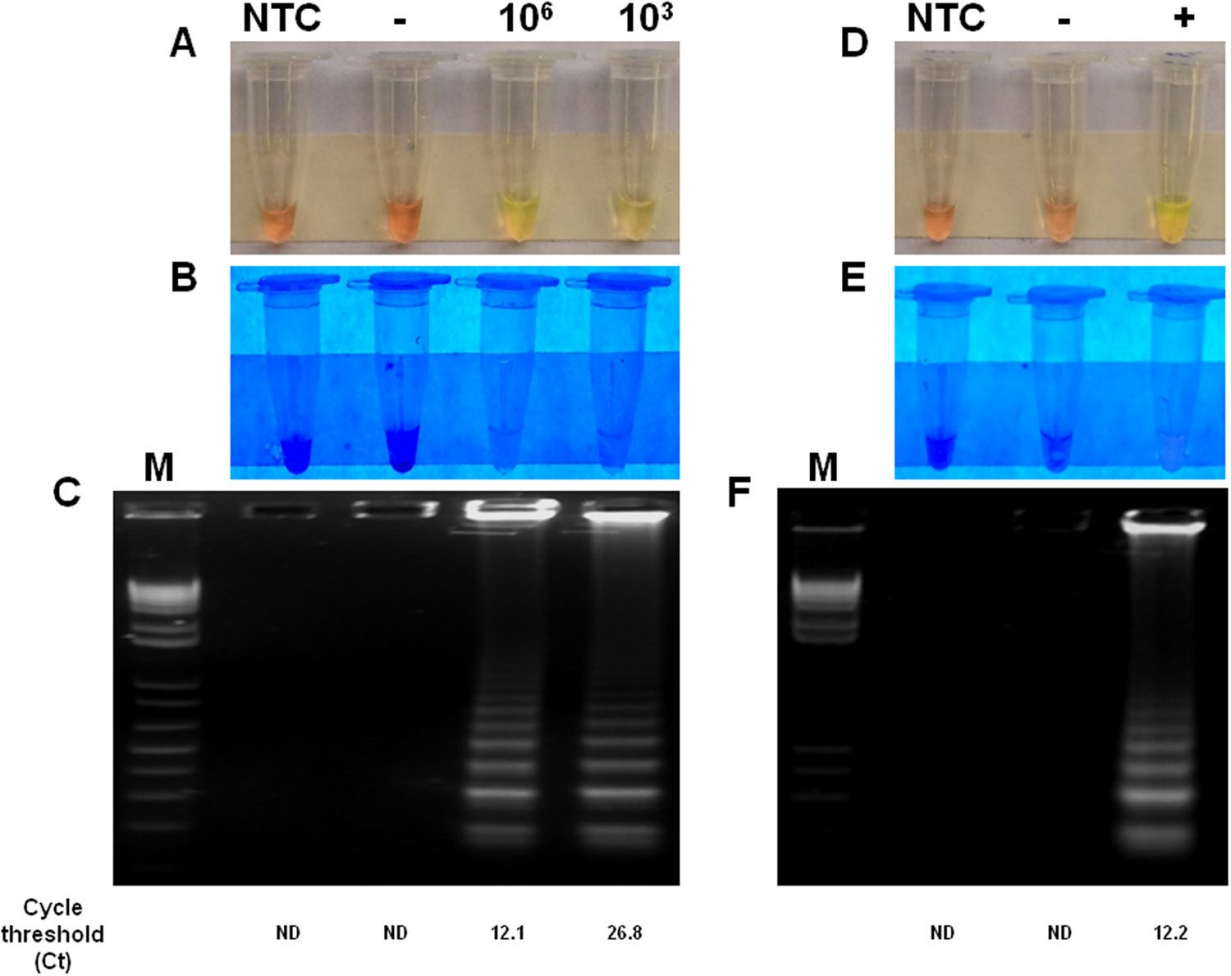
Detection of ZIKV in virus-spiked mosquito samples and crude lysate of experimentally infected *Aedes aegypti*. Crude lysates of uninfected *A*. *aegypti* were spiked with ZIKV to result in either a high (1 × 10^6^ PFU/mL) or low viral load (1 × 10^3^ PFU/mL) and processed for RT-LAMP without RNA isolation (**A**–**C**). (**D**–**F**) Represents RT-LAMP results of experimentally infected mosquitoes. RT-LAMP amplicons were observed by visual color change of the products and gel electrophoresis. The amplification products were observed by naked eye under natural light (**A**,**D**), under UV irradiation (**B**,**E**) and agarose gel electrophoresis (**C**,**F**). Legends in (**A**–**C**) are: NTC (non-template control): water; (−): macerate of uninfected *Aedes aegypti*; (10^6^): macerate of *Aedes aegypti* spiked with 10^6^ PFU; (10^3^): macerate of *Aedes aegypti* spiked with 10^3^ PFU. Legends in (**D**–**F**) are: NTC (non-template control): water; (−): macerate of uninfected *Aedes aegypti*; (+):macerate of *Aedes aegypti* experimentally infected with ZIKV. M: molecular weight marker.

First, we determined the ability of RT-LAMP to detect ZIKV in *A*. *aegypti* under controlled conditions. To this end, crude lysate of uninfected mosquitoes were spiked to result in either a high (1 × 10^6^ PFU/mL) or low viral load (1 × 10^3^ PFU/mL) in order to mimic physiological concentrations of ZIKV in these vectors. Spiked samples were processed for RT-LAMP without RNA isolation. RT-LAMP assay for ZIKV were positive in both viral loads tested. As expected, non template control (NTC) samples (water) and negative control (crude lysate of uninfected *A*. *aegypti*) tested negative (Fig. 1A–C). RNA extraction did not improve RT-LAMP detection (data not shown). RT-LAMP results were confirmed by qRT-PCR, through which the Ct value was 12.1 and 26.8, for high viral and low viral load, respectively (Figs 1 and S4). The same results were obtained with viral spike in *C*. *quinquefasciatus* homogenates (data not shown).

In order to mimic a real world scenario of ZIKV surveillance in mosquitoes, we determined the capacity of the RT-LAMP to detect ZIKV in *A*. *aegypti* mosquitoes experimentally infected by oral feeding on rabbit blood spiked with ZIKV. In this study, mosquitoes fed on unspiked rabbit blood were also included as controls. Crude mosquito lysates were used for RT-LAMP assay without RNA isolation. After incubation, the RT-LAMP was able to detect ZIKV only in infected mosquitoes, but not controls (Fig. 1D–F), suggesting the test may be useful for ZIKV detection in entomological samples. RNA extraction did not improve RT-LAMP detection (data not shown).

### Analytical specificity of RT-LAMP for detection of ZIKV

**Figure 2:**
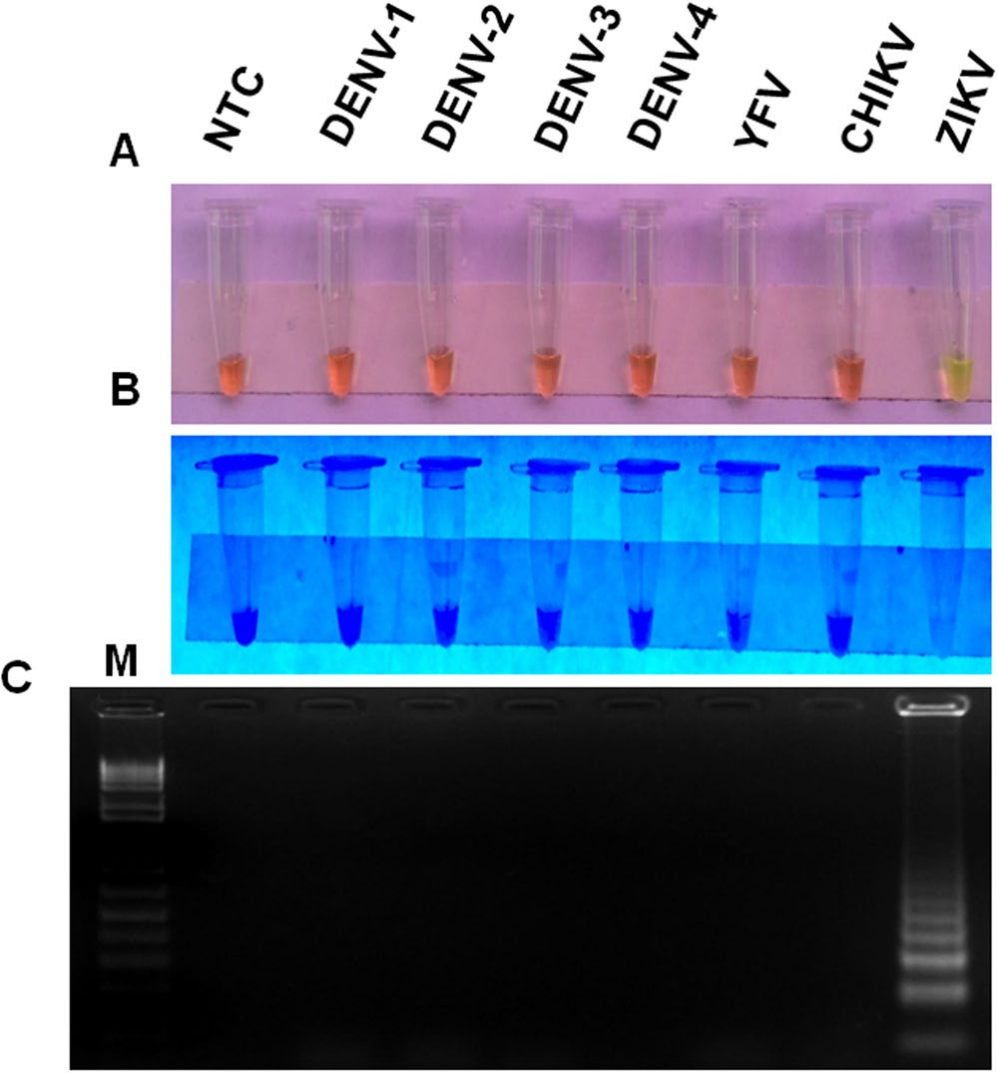
Analytical specificity of ZIKV RT-LAMP in mosquito samples. Crude lysates of uninfected *A*. *aegypti* were spiked with different arboviruses (DENV 1–4, YFV, CHIKV and ZIKV) circulating in Brazil so the final concentration would be 10^6^ PFU per reaction. Spiked samples were then assayed by ZIKV RT-LAMP. The amplification products were observed by naked eye under natural light (**A**), under UV irradiation (**B**) and agarose gel electrophoresis (**C**). M: molecular weight marker. NTC (non-template control): water.

To evaluate the specificity of the RT-LAMP assay to detect only ZIKV, we tested crude lysate of *A*. *aegypti* spiked with several arboviruses circulating in Brazil: DENV-1 (PE/97-42735), DENV-2 (PE/95-3808), DENV-3 (PE/02-95016), DENV-4 (PE/10-0081), YFV (17DD), and CHIKV (PE2016-480) (Table 1). Only the *A*. *aegypti* lysate spiked with ZIKV was positive in RT-LAMP reaction, as determined by naked eye analysis, visual observation under UV light or agarose gel electrophoresis (Fig. 2). Thus, these results suggested that RT-LAMP assay described here is highly specificity for detection of ZIKV.

**Table 1:** Viruses used in this study.

Family	Genus	Species	Strain	GenBank access code	Result of RT-LAMP
*Flaviviridae*	*Flavivirus*	Zika virus	PE-243	KX197192	+
		Dengue virus serotype 1	PE/97-42735	EU259529	−
		Dengue virus serotype 2	PE/95-3808	EU259569	−
		Dengue virus serotype 3	PE/02-95016	KC425219	−
		Dengue virus serotype 4	PE/10-0081	Unpublished	−
		Yellow fever virus	17DD	DQ100292	−
*Togaviridae*	*Alphavirus*	Chikungunya virus	PE2016-480	Unpublished	−

### Analytical sensitivity of RT-LAMP for detection of ZIKV

**Figure 3:**
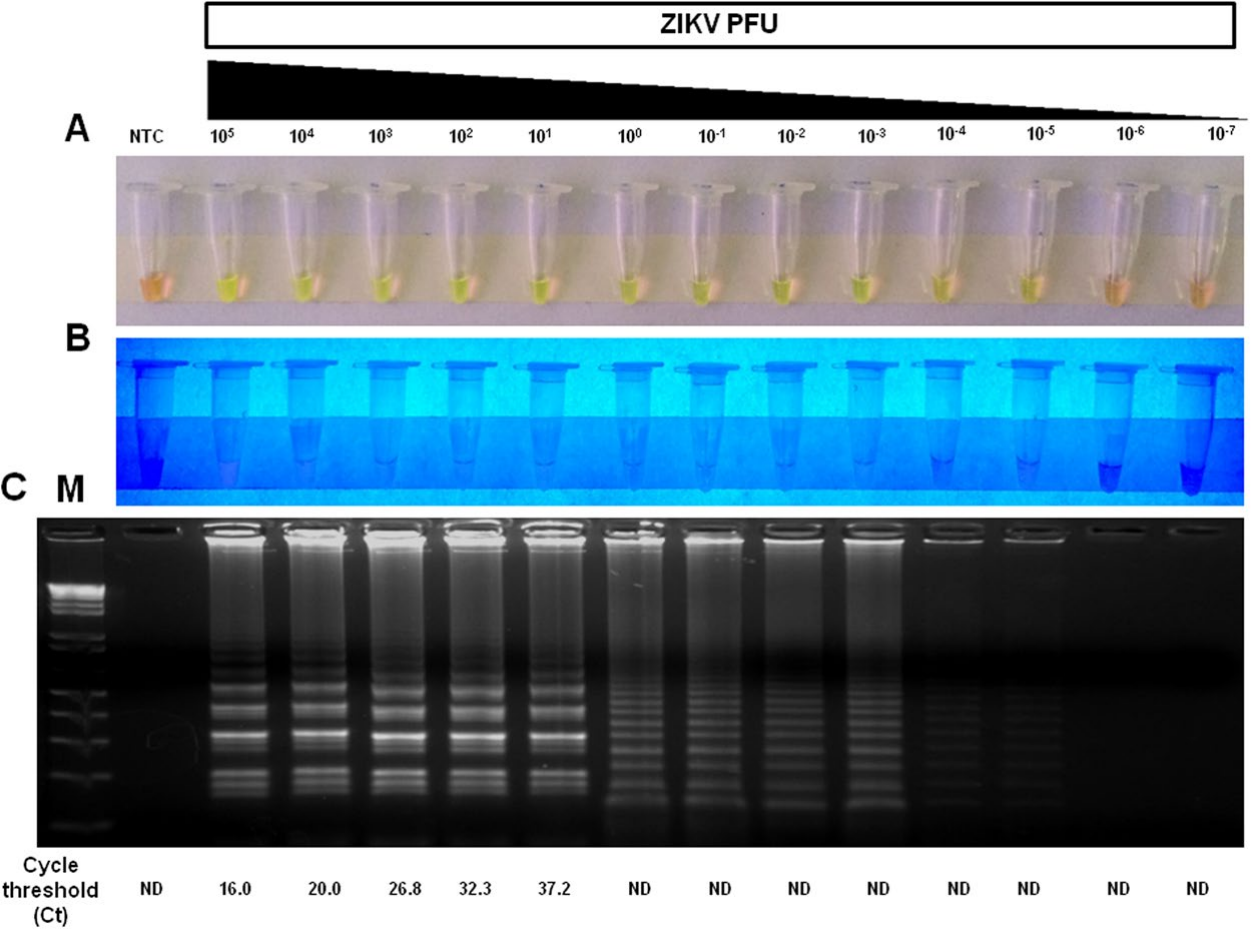
Analytical sensitivity of ZIKV RT-LAMP in mosquito samples. The sensitivity of RT-LAMP was determined by spiking different amounts (10^5^ PFU to 10^−7^ PFU) of ZIKV in crude lysates of uninfected *Aedes aegypti* mosquitoes and then testing by RT-LAMP directly without RNA extraction. The amplification products were observed by naked eye under natural light (**A**), under UV irradiation (**B**) and agarose gel electrophoresis (**C**). M: molecular weight marker. NTC (non-template control): water. ND (Not detected). To compare the results of RT-LAMP with a gold standard technique, viral RNA was extracted from the same dilutions using Trizol reagent and assayed qRT-PCR.

First, we sought to optimize the RT-LAMP assay conditions, reactions were performed at temperatures ranging from 59 °C to 75 °C following an incubation that ranged from 10 min to 60 min. The best amplification results were obtained at 72 °C for 40 min, but incubation time as short as 20 minutes was sufficient for detecting positive samples. Therefore, all assays were carried out using 40-min incubation time. The analytical sensitivity (limit of detection) of RT-LAMP was determined in crude lysate of *A*. *aegypti* spiked with a 10-fold serial dilution of ZIKV ranging from 10^5^ PFU to 10^−7^ PFU without RNA isolation. RT-LAMP was able to detect a broad range of virus concentration (from 10^5^ to 10^−5^ PFU), including viral loads found in naturally infected mosquitoes^39^. Considering 10 independent replicates per protocol developed, the probit regression analysis revealed that the limit of detection at 95% probability for each RT-LAMP was −2,98 log_10_ PFU of ZIKV (~1/1000 PFU) with confidence interval from −3,62 to −1,64 (Table 2 and Fig. S6). Additionally, viral RNA extracted from the same dilutions tested by RT-LAMP was assayed by the widely used ZIKV qRT-PCR method developed by Lanciotti^40^. For qRT-PCR assay, the lower detection limit was 10^1^ PFU ZIKV with Ct value 37.2 (Fig. 3). Taken together, the limit of detection was thus slightly than the gold standard technique for the diagnosis of ZIKV.

**Table 2:** Detection limit of the ZIKV RT-LAMP assay^a^.

ZIKV Concentration (PFU)	No. of Replicates	No. of positive results	Hit rate in %
10^5^	10	10	100
10^4^	10	10	100
10^3^	10	10	100
10^2^	10	10	100
10^1^	10	10	100
10^0^	10	10	100
10^−1^	10	10	100
10^−2^	10	10	100
10^−3^	10	9	90
10^−4^	10	7	70
10^−5^	10	6	60
10^−6^	10	0	0
10^−7^	10	0	0

^a^Probit regression analysis was calculated using MedCalc software (version 18.11), giving a C_95_ value (concentration detectable 95% of the time) of −2,98 log_10_ PFU of ZIKV. This indicates that the limit of detection is about −3 log_10_ (1/1000) PFU/reaction and that samples containing that concentration would be detected 95% of the time.

### Diagnostic performance of ZIKV RT-LAMP for mosquito samples

**Figure 4:**
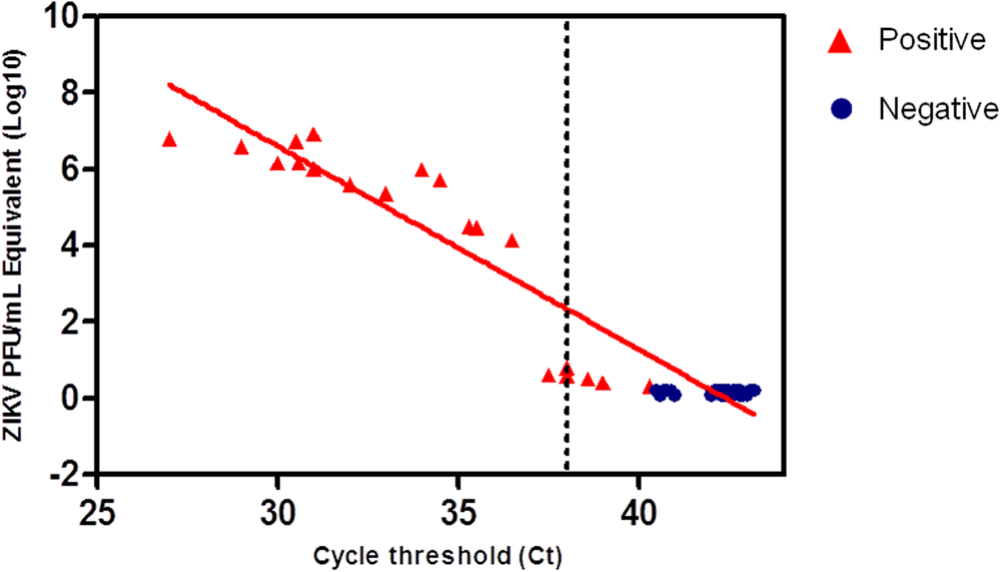
Diagnostic of mosquito samples by RT-LAMP. A total of 60 mosquito samples from *Aedes aegypti* (n = 32) and *Culex quinquefasciatus* (n = 28) were tested for ZIKV by RT-LAMP assay. Of these, 29 were positive for ZIKV and 31 were negative as determined by qRT-PCR. Dashed line represents the qRT-PCR cycle threshold (Ct value) value for ZIKV positivity (Ct ≤ 38). Red triangle indicates samples positive by RT-LAMP and blue circle are samples negative by RT-LAMP.

**Figure 5:**
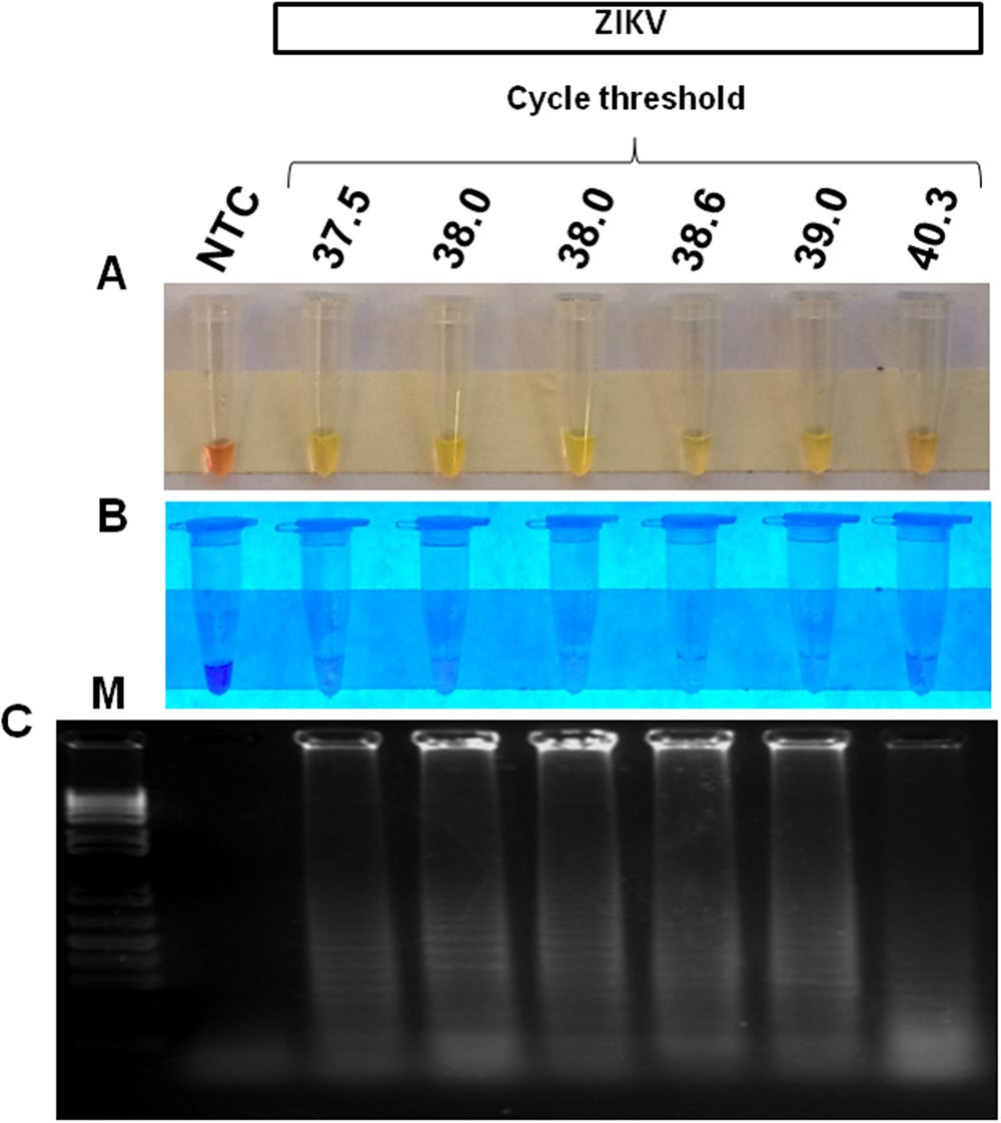
ZIKV detection by RT-LAMP in mosquito samples at the qRT-PCR detection limit. Mosquito samples at the detection threshold by qRT-PCR (Ct values ranging from 37.5 to 40.3) were assayed by RT-LAMP and all reactions showed a positive result. The amplification products were observed by naked eye under natural light (**A**), under UV irradiation (**B**) and agarose gel electrophoresis (**C**). M: molecular weight marker. NTC (non-template control): water.

A total of 60 mosquito samples from *A*. *aegypti* (n = 32) and *C*. *quinquefasciatus* (n = 28) were obtained from the Entomology Department^39,41^ and tested for ZIKV by RT-LAMP assay. Samples with Ct values of ≤38.0 in duplicate wells were considered positive for ZIKV infection^42^. Of these, 31 samples were ZIKV negative as determined by qRT-PCR and 29 were positive, including naturally and experimentally infected mosquitoes (Table 3). The Ct value in these samples ranged from 27.0 to >40.0. From the total of 60 samples, the RT-LAMP assay was able to detect ZIKV in 32 samples, including the 29 samples already determined to be positive by qRT-PCR (Fig. 4). Moreover, samples that were at the detection threshold by qRT-PCR (Ct values ranging from 37.5 to 40.3) were tested positive by the RT-LAMP assay result (Fig. 5), highlighting the sensitivity of the test in mosquito samples.

**Table 3:** Mosquito samples used for RT-LAMP validation.

Sample (ID)	Ct value	ZIKV PFU/mL equivalent	Mosquito sample	Source	Result of RT-LAMP
1	27.0	6.20 × 10^6^	*Culex quinquefasciatus*	Field sample	+
2	29.0	3.97 × 10^6^	*Culex quinquefasciatus*	Field sample	+
3	29.0	3.97 × 10^6^	*Aedes aegypti*	Laboratory sample	+
4	30.0	1.50 × 10^6^	*Culex quinquefasciatus*	Field sample	+
5	30.0	1.50 × 10^6^	*Culex quinquefasciatus*	Field sample	+
6	30.0	1.50 × 10^6^	*Aedes aegypti*	Laboratory sample	+
7	30.5	5.31 × 10^6^	*Aedes aegypti*	Field sample	+
8	30.6	1.45 × 10^6^	*Aedes aegypti*	Laboratory sample	+
9	30.6	1.45 × 10^6^	*Aedes aegypti*	Laboratory sample	+
10	31.0	8.23 × 10^6^	*Aedes aegypti*	Field sample	+
11	31.0	8.23 × 10^6^	*Aedes aegypti*	Field sample	+
12	31.0	8.23 × 10^6^	*Culex quinquefasciatus*	Field sample	+
13	31.0	8.23 × 10^6^	*Aedes aegypti*	Laboratory sample	+
14	32.0	3.91 × 10^5^	*Aedes aegypti*	Field sample	+
15	32.0	3.91 × 10^5^	*Aedes aegypti*	Field sample	+
16	32.0	3.91 × 10^5^	*Culex quinquefasciatus*	Field sample	+
17	32.0	3.91 × 10^5^	*Culex quinquefasciatus*	Field sample	+
18	33.0	2.27 × 10^5^	*Culex quinquefasciatus*	Field sample	+
19	34.0	9.97 × 10^5^	*Aedes aegypti*	Field sample	+
20	34.0	9.97 × 10^5^	*Aedes aegypti*	Field sample	+
21	34.5	5.17 × 10^5^	*Aedes aegypti*	Field sample	+
22	35.3	3.23 × 10^4^	*Aedes aegypti*	Field sample	+
23	35.5	3.00 × 10^4^	*Aedes aegypti*	Field sample	+
24	35.5	3.00 × 10^4^	*Aedes aegypti*	Field sample	+
25	36.5	1.41 × 10^4^	*Aedes aegypti*	Field sample	+
26	36.5	1.41 × 10^4^	*Aedes aegypti*	Field sample	+
27	37.5	6.00 × 10^1^	*Culex quinquefasciatus*	Field sample	+
28	38.0	5.60 × 10^1^	*Aedes aegypti*	Field sample	+
29	38.0	5.60 × 10^1^	*Aedes aegypti*	Field sample	+
30	38.6	5.00 × 10^1^	*Aedes aegypti*	Field sample	+
31	39.0	4.15 × 10^1^	*Culex quinquefasciatus*	Field sample	+
32	40.3	0	*Culex quinquefasciatus*	Field sample	+
33	>40.0	0	*Aedes aegypti*	Field sample	−
34	>40.0	0	*Aedes aegypti*	Field sample	−
35	>40.0	0	*Aedes aegypti*	Field sample	−
36	>40.0	0	*Aedes aegypti*	Field sample	−
37	>40.0	0	*Aedes aegypti*	Field sample	−
38	>40.0	0	*Culex quinquefasciatus*	Field sample	−
39	>40.0	0	*Culex quinquefasciatus*	Field sample	−
40	>40.0	0	*Culex quinquefasciatus*	Field sample	−
41	>40.0	0	*Culex quinquefasciatus*	Field sample	−
42	>40.0	0	*Culex quinquefasciatus*	Field sample	−
43	>40.0	0	*Culex quinquefasciatus*	Field sample	−
44	>40.0	0	*Culex quinquefasciatus*	Field sample	−
45	>40.0	0	*Culex quinquefasciatus*	Field sample	−
46	>40.0	0	*Culex quinquefasciatus*	Field sample	−
47	>40.0	0	*Culex quinquefasciatus*	Field sample	−
48	>40.0	0	*Culex quinquefasciatus*	Field sample	−
49	>40.0	0	*Culex quinquefasciatus*	Field sample	−
50	>40.0	0	*Culex quinquefasciatus*	Field sample	−
51	>40.0	0	*Culex quinquefasciatus*	Field sample	−
52	>40.0	0	*Culex quinquefasciatus*	Field sample	−
53	>40.0	0	*Culex quinquefasciatus*	Field sample	−
54	>40.0	0	*Culex quinquefasciatus*	Field sample	−
55	>40.0	0	*Culex quinquefasciatus*	Field sample	−
56	>40.0	0	*Aedes aegypti*	Laboratory sample	−
57	>40.0	0	*Aedes aegypti*	Laboratory sample	−
58	>40.0	0	*Aedes aegypti*	Laboratory sample	−
59	>40.0	0	*Aedes aegypti*	Laboratory sample	−
60	>40.0	0	*Aedes aegypti*	Laboratory sample	−

The diagnostic performance of ZIKV RT-LAMP for mosquito samples was determined by statistical analysis using qRT-PCR as the gold standard technique. The overall ZIKV prevalence in the samples was 46.03% (95% CI 33.39% to 59.06%). The RT-LAMP assay had a diagnostic sensitivity of 100% (95% CI 88.06% to 100.00%) and diagnostic specificity of 91.18% (95% CI 76.32% to 98.14%). The positive predictive value, which is probability that the virus is present when the test is positive, was 90.62% (95% CI 76.64% to 96.61%), whereas the negative predictive value, which indicates the probability that the virus is absent when the test is negative, was 100%. The overall accuracy of the RT-LAMP test was determined to 95.24% (95% CI 86.71% to 99.01%) (Table 4), highlighting the practical value of RT-LAMP for ZIKV detection in entomological samples.

**Table 4:** Diagnostic performance of ZIKV RT-LAMP for mosquito samples.

	qRT-PCR +	qRT-PCR −	Total
RT-LAMP +	29	3	32
RT-LAMP −	0	31	31
Total	29	34	
Sensitivity	**100%** (95% CI 88.06% to 100.00%)		
Specificity	**91.18%** (95% CI 76.32% to 98.14%)		
ZIKV prevalence	**46.03%** (95% CI 33.39% to 59.06%)		
Positive Predictive Value	**90.62%** (95% CI 76.64% to 96.61%)		
Negative Predictive Value	**100%**		
Accuracy	**95.24%** (95% CI 86.71% to 99.01%)		

**Figure 6:**
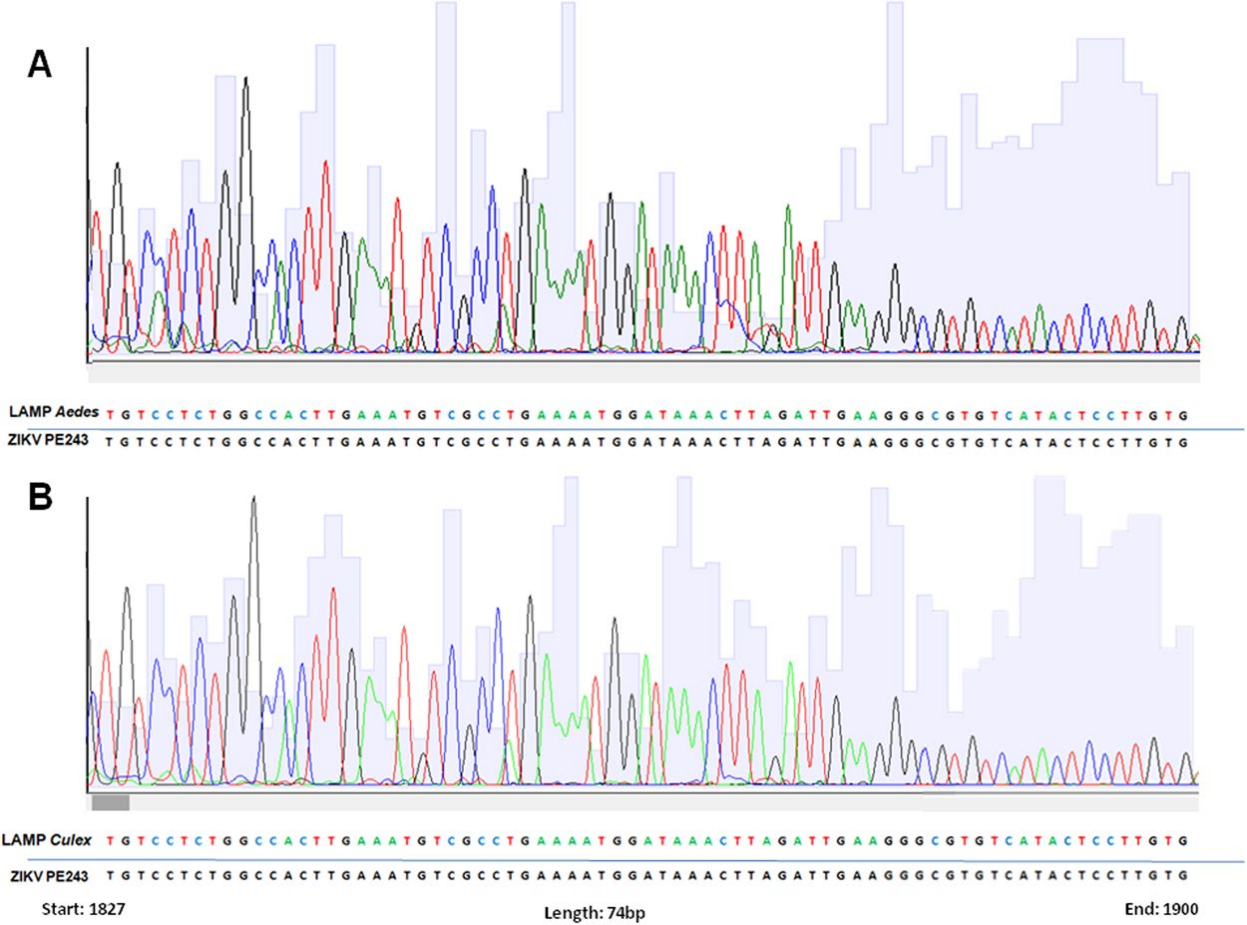
Electropherogram of ZIKV RT-LAMP detected in field-caught *Aedes spp*. and *Culex spp*. mosquitoes. Amplicons from RT-LAMP reaction from field-caught *Aedes aegypti* (**A**) and *Culex quinquefasciatus* mosquitoes (**B**) were sequenced using Sanger method to confirm the identity of ZIKV. The region amplified was genome position 1827 to 1900. The obtained sequences were aligned against the ZIKV PE243 reference strain.

To confirm the identity of ZIKV RT-LAMP positive samples, we sequenced positive samples from field-caught *Aedes spp*. and *Culex spp*. mosquitoes by the Sanger method. Sequencing results and BLAST analysis demonstrated that ZIKV RT-LAMP amplicons match 100% with virus circulating in Brazil (Fig. 6), confirming the specificity of the RT-LAMP for ZIKV.

Together, these results indicated that our ZIKV RT-LAMP assay represents a robust and affordable diagnostic platform that can be used as a surveillance tool for mosquitoes infected with ZIKV.

## Discussion

The rapid detection of ZIKV in mosquito samples can help to understand the dynamics of the disease in areas that have favorable conditions for virus transmission^20^. In this context, we developed a rapid molecular test for the detection of ZIKV in mosquito samples that may be a valuable tool for vector surveillance. The RT-LAMP assay described here is straightforward, inexpensive, and enables ZIKV detection even in the absence of RNA extraction. To our knowledge, this is the first validation of a ZIKV RT-LAMP assay using experimentally and naturally infected *A*. *aegypti* and *C*. *quinquefasciatus* mosquitoes collected at the epicenter of the Zika epidemic in Brazil.

Currently, the gold standard technique for detection of ZIKV in mosquito samples is qRT-PCR. This assay is specific for detecting the virus in both human and mosquito samples^21,40^. However, its prohibitive cost makes qRT-PCR unfit for testing a large number of mosquitoes collected in entomological surveillance programs^41^. Another potential limitation of qRT-PCR is the inability to detect low viral titers, which may occur especially during interepidemic periods. The limit of detection for the assay described by Faye was 0.05 plaque forming unit (PFU) or 32 genome-equivalents and the one developed by Lanciotti was 25 RNA copies^21,40^. Recently, other research groups have developed methodologies using the LAMP approach for the detection of ZIKV using mosquito samples^34–36^. However, these studies used only a handful of mosquito samples and the lowest virus concentration detected was 10^3^ PFU. Our RT-LAMP was evaluated using 60 and revealed to be about 10,000 fold more sensitive than the qRT-PCR, detecting virus concentrations as low as 10^−5^ PFU. The large amount of infectious and non-infectious ZIKV RNA released into the culture supernatant explains the ability of RT-LAMP to detect less than 1 PFU even without RNA extraction^43^. The analytical sensitivity of both our qRT-PCR and RT-LAMP differed from previously published studies which developed the primers^30,40^. There are a number of reasons that might have accounted for this variation, including differences in kits and research suppliers, viral strains, type of biological samples, and detection systems.

Several mosquito-borne arboviruses, including ZIKV, DENV and CHIKV, are endemic and co-circulate throughout the Northeast Brazil^44,45^. One possible limitation of diagnostic tests for ZIKV is the possibility of cross-reactivity with other flaviviruses, particularly DENV^40,46,47^. Here, we showed no cross-reactions with other arboviruses including four serotypes of DENV, YFV or CHIKV and sequencing of RT-LAMP amplicons from naturally infected *A*. *aegypti* and *C*. *quinquefasciatus* confirmed ZIKV identity.

We validated the RT-LAMP assay using samples obtained from experimentally and naturally ZIKV-infected *A*. *aegypti* and *C*. *quinquefasciatus*. The RT-LAMP had a sensitivity of 100%, specificity of 91.18%, and overall accuracy of 95.24% as compared to qRT-PCR. Importantly, the ZIKV RT-LAMP could undoubtedly detect ZIKV RNA in mosquito samples that had been previously tested as negative by qRT-PCR. These samples were at the detection threshold by the qRT-PCR with Ct value ranging from 38.6 to 40.3. In contrast with our findings, some studies have reported that the analytical sensitivity of the RT-LAMP assay is lower when compared to the gold standard diagnostic test (qRT-PCR)^32,38^. However, recently published studies have corroborated our findings that the analytical sensitivity of the RT-LAMP assay is superior than qRT-PCR^36,48^.

The RT-LAMP assay can be performed through either a two-step assay or one-step protocol. Two-step RT-LAMP requires the addition of the reverse transcriptase (RT) enzyme together with the DNA polymerase enzyme, which may be wild-type Bst DNA polymerase or Bst 2.0 polymerase 2.0 WarmStart. Several studies report the need for RNA extraction before performing the RT-LAMP assay and the use of the two-step RT-LAMP^49–51^. However, the two step protocol is longer, more expensive, and requires additional sample handling, which increases the chances of pipetting errors and contamination. The use of Bst 3.0 Polymerase 3.0 WarmStart overcomes these concerns. This enzyme possesses high activity of reverse transcriptase and polymerase in a single-temperature incubation which allows the assay to be performed in a one-step. Additionally, the Bst 3.0 DNA polymerase is a robust enzyme capable of maintaining its activities even in the presence of inhibitors^33^. This is especially relevant for viral survey in entomological samples which are notorious to harbor amplification inhibitors^52^.

Recently, Yaren *et al*. reported a diagnostic test based on RT-LAMP for detection of ZIKV in mosquito samples^35^. Nonetheless, the need for RNA extraction limits its applications for POC diagnostics. In another study, Lamb *et al*. reported a low-cost molecular diagnostic test method based on RT-LAMP for detection of ZIKV in mosquito samples without RNA isolation^36^. However, the authors tested only five experimentally infected *A*. *aegypti* and did not validate the technique using naturally infected mosquitoes.

Other groups have also developed several technologies for molecular detection of ZIKV^30–33,35,50,51,53–58^. However, many of these technologies still have limitations for POC diagnostic applications, including the need for RNA isolation or the use of sophisticated and proprietary hardware and software, which limits its applicability in the developing world.

The main advantages of the RT-LAMP assay described here is the ability to detect ZIKV without the need for pretreatment or RNA extraction from the mosquito samples. Importantly, positive samples can be diagnosed in just 20 minutes and the result can be easily interpreted visual examination. Given its simplicity, the assay can be run by individuals without specialty training. The cost per sample was inferior to $1, which is considerably lower than qRT-PCR. These advantages suggest that our diagnostic assay to detect ZIKV is suitable for use in viral surveillance in mosquitoes in remote areas or low resource countries affected by the ZIKV epidemics or at risk of viral introduction.

## Conclusion

We have developed a low cost, point-of-care diagnostic platform based on the RT-LAMP assay to detect ZIKV in mosquito samples collected at the epicenter of the Zika epidemics in Brazil. The test is a robust, fast and inexpensive tool for surveillance of ZIKV in mosquito populations and will enable developing countries to establish better viral surveillance in vectors and improve the efficacy of control programs. Our results provide a potential new molecular diagnostic test for ZIKV in mosquito samples as a novel straightforward and inexpensive method for detection of ZIKV in arthropod vectors.

## Methods

### Cells and viruses

Vero cells were grown in Dulbecco’s modified Eagle’s medium (DMEM) (Gibco, Carlsbad, CA) supplemented with 10% inactivated fetal bovine serum (FBS) (Gibco), 2 mM l-glutamine (Gibco) and 100 U/mL penicillin/streptomycin (Gibco) at 37 °C in 5% CO_2_. The ZIKV strain PE243 (GenBank access code: KX197192.1) used in this work was isolated in C6/36 cell line using serum sample of a Brazilian patient infected by ZIKV in 2015. After isolation, the virus was propagated and stored at −80 °C until use. Other arboviruses, including DENV-1 (PE/97-42735), DENV-2 (PE/95-3808), DENV-3 (PE/02-95016), DENV-4 (PE/10-0081), YFV (17DD) and CHIKV (PE2016-480) were similarly propagated in Vero cells and used to determine the specificity of the RT-LAMP. All viruses were titrated in Vero cells by the standard plaque assay method and resulted in titers ranging from 10^6^ to 10^7^ PFU/mL. With the exception of YFV (17DD), which is a vaccine strain, all other viruses were isolated from humans in Pernambuco, Brazil.

### RT-LAMP assay

RT-LAMP reactions were carried out in triplicate in a total volume of 25 μL containing 1x Isothermic Amplification Buffer, 8 mM MgSO4, 4 U of Bst DNA polymerase [version 3.0 WarmStart; New England Biolabs (NEB)], 1.8 mM deoxynucleotide triphosphates (dNTPs) (ThermoFisher Scientific), 1.6 μM for FIP (5′-GGCGACATTTCAAGTGGCCAGAGAGCTCTRGAGGCTGAGA-3′), 1.6 μM for BIP (5′-AGGGCGTGTCATACTCCTTGTGAGTGTTTCAGCCGGGATCT-3′), 0.2 μM for F3 (5′-CAGTTCACACGGCCCTTG-3′), 0.2 μM for B3 (5′-TGTACCTCCACTGTGACTGT-3′), 0.4 μM for LF (5′-CCTTCCCTTTGCACCATCCA-3′), 0.4 μM for LB (5′-TACCGCAGCGTTCACATTCA) primers and 5 μL test sample (no template control (NTC), extracted RNA, or samples without RNA extraction). Theses primers have been previously described^30^. In order to visualize positive reactions and prevent contamination, 1 μL of SYBR Green I (ThermoFisher Scientific) diluted 1:10 dilution in RNase-free water (Promega) was added to the center of the tube caps before the reaction and mixing afterwards. Reactions were incubated at 72 °C for 40 min in a heat block, and then inactivated at 80 °C for 5 minutes. To evaluated the robustness of the assay for POC applications, all set-up and execution of RT-LAMP reactions were done in a conventional lab bench using designated pipettes and filter tips. Imaging analysis took place in separate rooms. All experiments were independently replicated at least six times.

After the incubation, the RT-LAMP products reactions were detected using three different methods. In the first, the products were observed by naked eye under natural light and photographed using a conventional smartphone camera. A color change from orange to greenish yellow was used to identify positive sample, while a negative sample remained orange. The second method was visual analysis of reaction tubes under UV light irradiation (UV wavelength of 302–312 nm) using a transilluminator (model UVB LTB 20 × 20 STV, Loccus Biotecnologia, São Paulo, Brazil) coupled with a camera and connected to a computer. In this method, negative samples were dark blue and positive reactions were light fluorescent. In the third method, the RT-LAMP amplicons were analyzed by agarose gel electrophoresis (2.0%) in 1x TAE buffer, followed by ethidium bromide staining and gel visualization using transilluminator. For electrophoresis analysis, 1 kb Plus DNA Ladder (ThermoFisher Scientific) was used as a DNA size marker.

### Real time RT-PCR

Samples with ZIKV are tested for positivity of the infection by qRT-PCR, according to protocols established by the Centers for Disease Control and Prevention - CDC USA with minor modifications^40^. Briefly, RNA from samples was extracted using Trizol reagent (Invitrogen Carlsbad, USA) following the instructions of the manufacturer. qRT-PCR was conducted using the QuantiNova Probe RT-PCR Kit (QIAGEN, Valencia, CA, USA) with amplification in the Applied Biosystems 7500 real-time PCR system (Applied Biosystems, Foster City, CA, USA) as per the manufacturer’s protocol. The reaction mixture (total volume, 15 μL) contained 7.5 μL of QuantiNova Probe RT-PCR Master Mix 2× , 0.9 μM each primers Zika1087 (5′-CCGCTGCCCAACACAAG-3′), Zika1163C (5′-CCACTAACGTTCTTTTGCAGACAT-3′), 0.9 μM FAM-labelled 1108 (5′-AGCCTACCTTGACAAGCAGTCAGACACTCAA-3′) probe for ZIKV, 0.1 μL of QuantiNova RT Mix, 0.08 μL of QuantiNova ROX Reference Dye, 5 μL of the RNA samples and RNA-free water. Primers and probes were synthesized by IDT (Integrated DNA Technologies, Skokie, Illinois, USA). The reaction program consisted of a single cycle of reverse transcription for 15 min at 45 °C, followed by 5 min at 95 °C for reverse transcriptase inactivation and DNA polymerase activation, and then 45 cycles of 5 s at 95 °C and 45 s at 60 °C. The amount of viral RNA in each sample was estimated by comparing the cycle threshold values (Ct) to the standard curve made by serial 10-fold serial dilutions of previously titrated ZIKV BRPE243/2015.

### Detection of ZIKV in Mosquito Samples Under Controlled Conditions

To evaluate the ability of RT-LAMP to detect ZIKV in mosquitoes, pools of *A*. *aegypti* or *C*. *quinquefasciatus* mosquitoes (n = 10) were homogenized in 300 μL of RNA-free water. Crude lysates were then spiked with 100 µL of ZIKV so the final viral concentration in the lysates was either 10^6^ or 10^3^ PFU/mL, thus simulating a situation of high and low viral load, respectively. After incubation at 37 °C for 1 hour, samples were directly assay by RT-LAMP without RNA extraction.

In order to assess ZIKV detection by RT-LAMP in infected mosquitoes, we used samples from experimentally infected female *A*. *aegypti* mosquitoes. In brief, the Rec-Lab colony was maintained under standard conditions (temperature, 26 °C ± 1 °C, relative humidity of 60 to 80% and photoperiod 12:12 h C/E) at the Entomology Laboratory of the Institute Aggeu Magalhães (IAM). For artificial feeding, cell supernatant containing 10^6^ PFU of ZIKV were mixed in 1:1 defibrinated rabbit blood and provided to starving mosquitoes for for 90 minutes as previously described^39^. Whole female mosquitoes were collected at 18 days post-infection, homogenized in 300 μL of RNA-free and processed for RT-LAMP. Mosquitoes independently fed on non-infected culture cells mixed to the defibrinated rabbit blood was used as controls.

### Analytical Specificity and Analytical Sensitivity of RT-LAMP

To test specificity of the RT-LAMP primers for ZIKV, primers were validated by testing the cross-reactivity with other arboviruses currently circulating in Brazil, including ZIKV (PE243), four different serotypes of dengue DENV-1 (PE/97-42735), DENV-2 (PE/95-3808), DENV-3 (PE/02-95016), DENV-4 (PE/10-0081), YFV (17DD) and CHIKV (PE2016-480). Crude lysates of uninfected *A*. *aegypti* were spiked with different arboviruses so the final concentration would be 10^6^ PFU per reaction. Spiked samples were then assayed by ZIKV RT-LAMP.

To evaluate the analytical sensitivity (limit of detection) of the RT-LAMP assay, ZIKV strain PE243 was 10-fold serially diluted in crude lysates of uninfected *A*. *aegypti* mosquito. Virus concentration in spiked mosquito samples ranged from 10^5^ PFU to 10^−7^ PFU. After dilution, samples were directly assayed by RT-LAMP without RNA isolation. To compare the results of RT-LAMP with a gold standard technique, viral RNA was extracted from the same dilutions using Trizol reagent (Invitrogen Carlsbad, USA) according the manufacturer’s instructions and then assayed by the widely used ZIKV qRT-PCR method^40^.

### Validation of RT-LAMP for ZIKV detection in Mosquito Samples

To validate the performance of the RT-LAMP for the diagnosis of ZIKV relative to qRT-PCR, 60 samples from *A*. *aegypti* (n = 32) and *C*. *quinquefasciatus* (n = 28) previously assayed by qRT-PCR^11,41^ were obtained from the Entomology Department and tested by RT-LAMP. The intrinsic diagnostic utility of the test was determined using several statistical parameters described below.

### Sequencing of LAMP fragments

The genetic characterization of the LAMP fragments from two field positives samples from *A*. *aegypti* and *C*. *quinquefasciatus* was performed by the Sanger sequencing method. Amplicons from RT-LAMP reaction were directly purified using illustra GFX PCR DNA and Gel Band Purification Kit (GE) according to the manufacturer´s instructions and eluted in 30 μL of water. Purified amplicons were directly sequenced using the primer FIP and the BigDye Terminator v3.1 Cycle Sequencing Kit (Applied Biosystems,USA) as established by the manufacturer and run on an ABI Prism 3100 Capillary Automatic DNA Analyzer. Sequences of fragments were analyzed using the Bioedit software, v7.0.5 and submitted to NCBI BLAST database (http://www.ncbi.nlm.nih.gov/blast/Blast.cgi) to identify the most closely ZIKV strain.

### Statistical analysis

Graphs were generated using the GraphPad Prism Software version 5.01 for Windows (GraphPad Software, La Jolla, California, USA). A probit regression was performed to calculate the limit of detection of the RT-LAMP for detection of ZIKV using MedCalc software (version 18.11, MedCalc Software, Ostend, Belgium). The estimation of the several diagnostic parameters (sensitivity, specificity, ZIKV prevalence, positive predictive value, negative predictive value and overall accuracy) of the RT-LAMP for detection of ZIKV was calculated using the web-based software MedCalc’s Diagnostic Test Evaluation Calculator (https://www.medcalc.org/calc/diagnostic_test.php). This analysis was based on the results from 60 mosquito samples previously diagnosed by qRT-PCR.

## Supplementary information

Dataset 1
