# Supplementary material for: Development and Validation of Reverse Transcription Loop-Mediated Isothermal Amplification (RT-LAMP) for Rapid Detection of ZIKV in Mosquito Samples from Brazil
Source: Sci Rep. 2019 Mar 14;9:4494. doi: 10.1038/s41598-019-40960-5 (PMC6418238; doi:10.1038/s41598-019-40960-5)
Supplement: Supplementary file 1 — Dataset 1 [file 41598_2019_40960_MOESM1_ESM.docx]

**Supplementary Information**

**Development and Validation of Reverse Transcription Loop-Mediated Isothermal Amplification (RT-LAMP) for Rapid Detection of ZIKV in Mosquito Samples from Brazil**

Severino Jefferson Ribeiro da Silva^1^, Marcelo Henrique Santos Paiva^2,3^, Duschinka Ribeiro Duarte Guedes^3^, Larissa Krokovsky^3^, Fábio Melo^4^, Maria Almerice Lopes da Silva^4^, Adalúcia Silva^1^, Constância Flávia Junqueira Ayres^3^, Lindomar J. Pena^1*^

^1^Department of Virology, Oswaldo Cruz Foundation (Fiocruz), Recife, Pernambuco, Brazil;

^2^Agreste Academic Center, Federal University of Pernambuco (UFPE), Caruaru, Pernambuco, Brazil;

^3^Department of Entomology, Oswaldo Cruz Foundation (Fiocruz), Recife, Pernambuco, Brazil;

^4^Department of Parasitology, Oswaldo Cruz Foundation (Fiocruz), Recife, Pernambuco, Brazil;

*Corresponding author:

Lindomar Pena, PhD. Department of Virology, Oswaldo Cruz Foundation (Fiocruz). Address: Avenida Professor Moraes Rego. Recife, Pernambuco, Brazil. Email: [lindomar.pena@cpqam.fiocruz.br](mailto:lindomar.pena@cpqam.fiocruz.br)

**Figure S1. Original images of the gels shown in Figure 1.
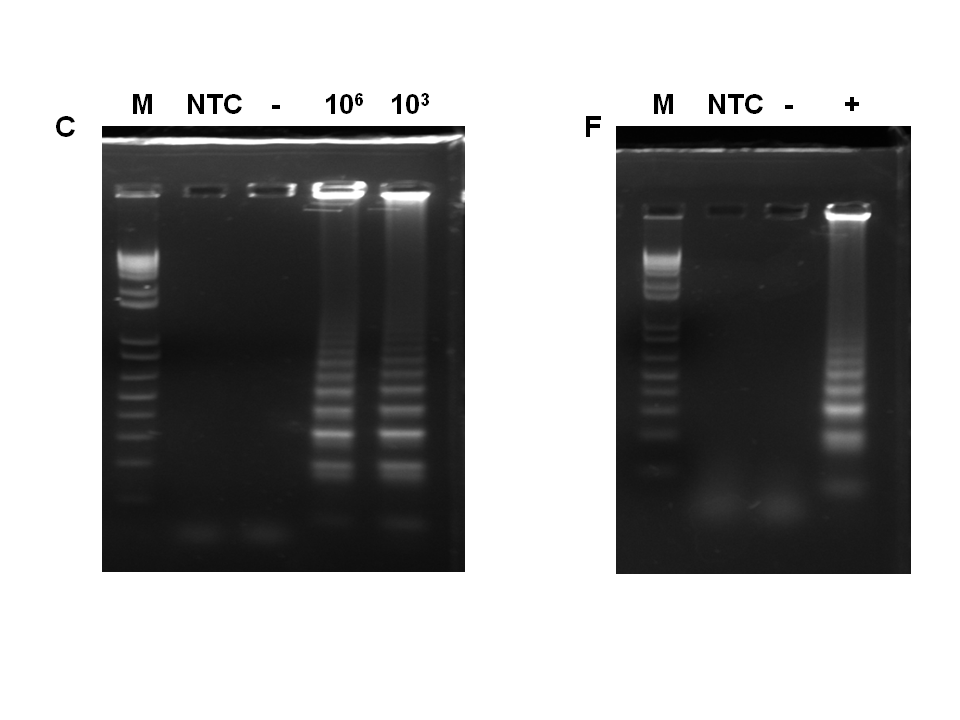
**

**Figure S2. Original image of the gel shown in Figure 2.**

**
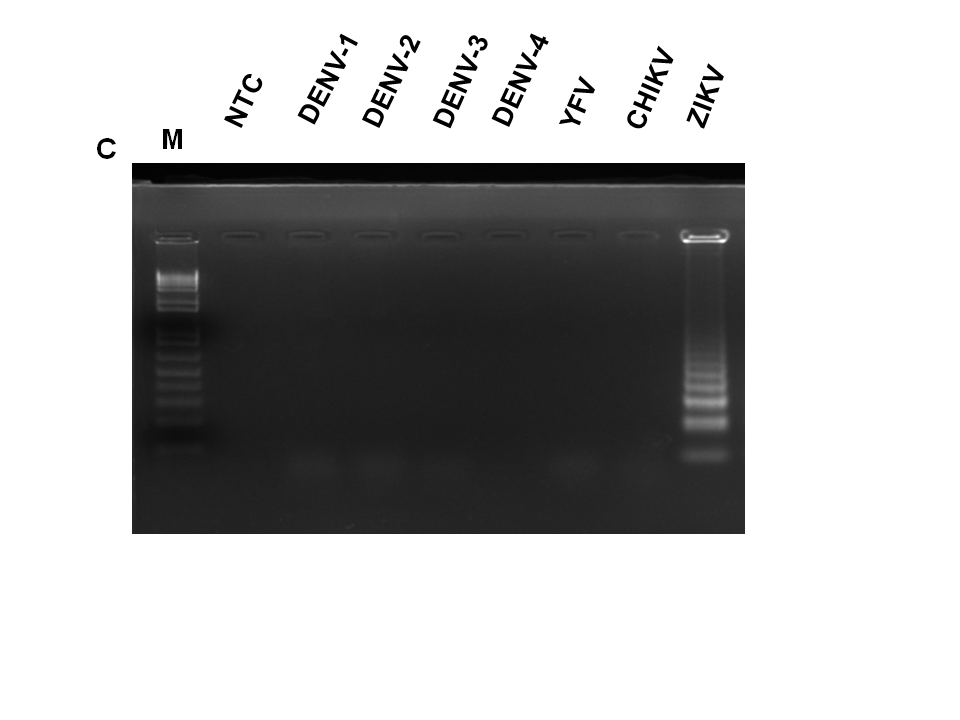
**

**Figure S3. Original image of the gel shown in Figure 3.**

**
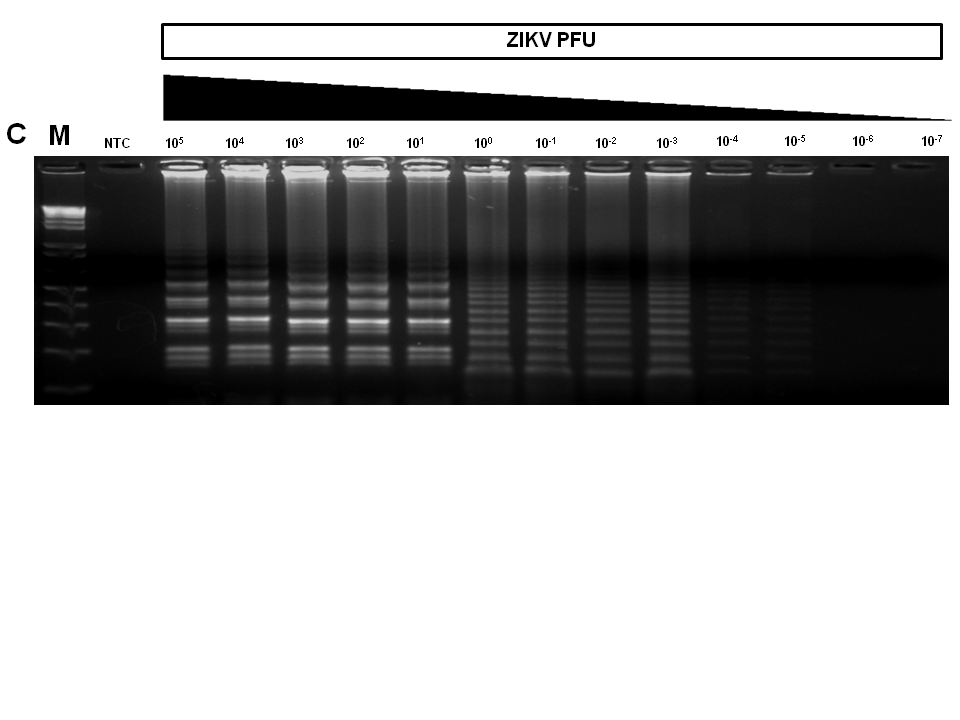
**

**Figure S4. Amplification plots obtained from the qRT-PCR.** RT-LAMP results were confirmed by qRT-PCR with the infected lysates, through which the Ct value was 12.1 and 26.8, for high viral and low viral load, respectively.

**
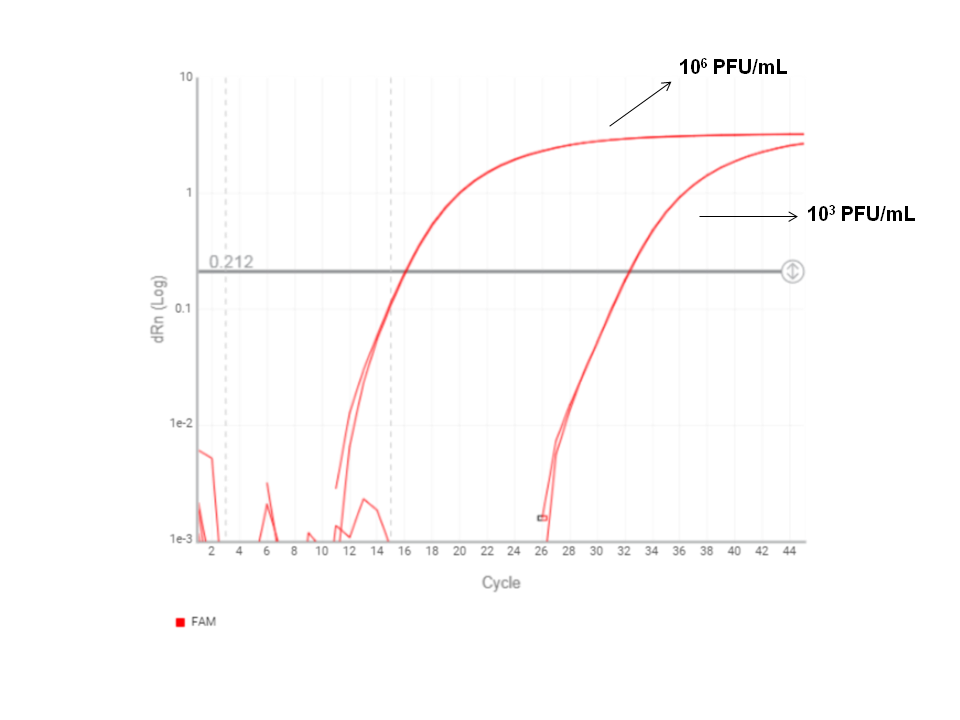
**

**Figure S5. Original image of the gel shown in Figure 5.**

**
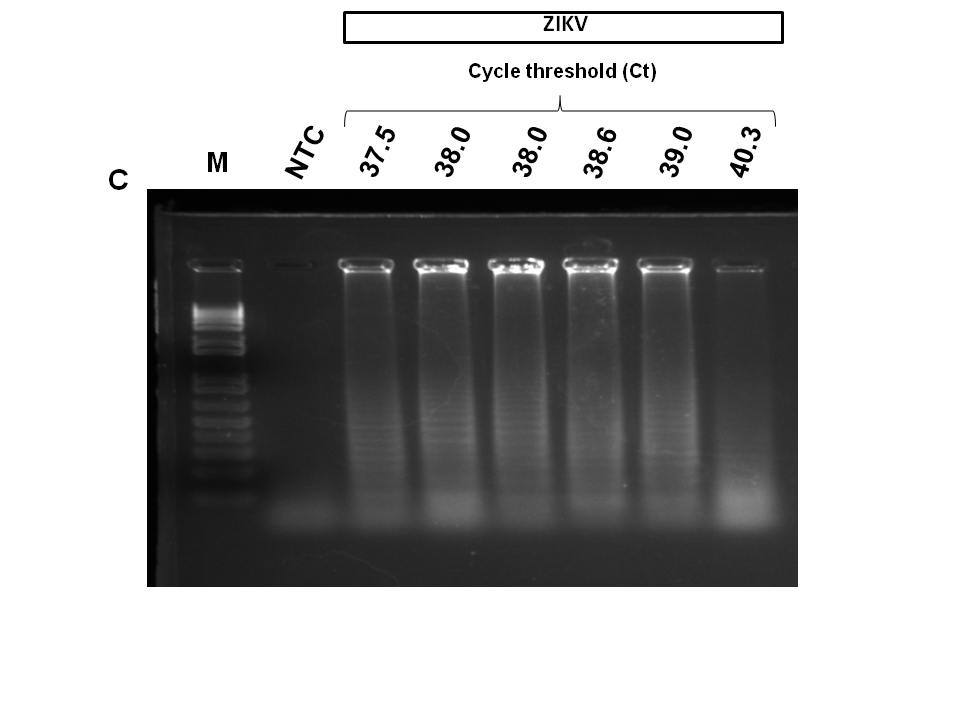
**

**Figure S6. Limit of detection of the ZIKV RT-LAMP assay.** The probit regression analysis curve was obtained from ten replicates of serial dilutions (10^5^ – 10^-7^ PFU) using MedCalc software.

**
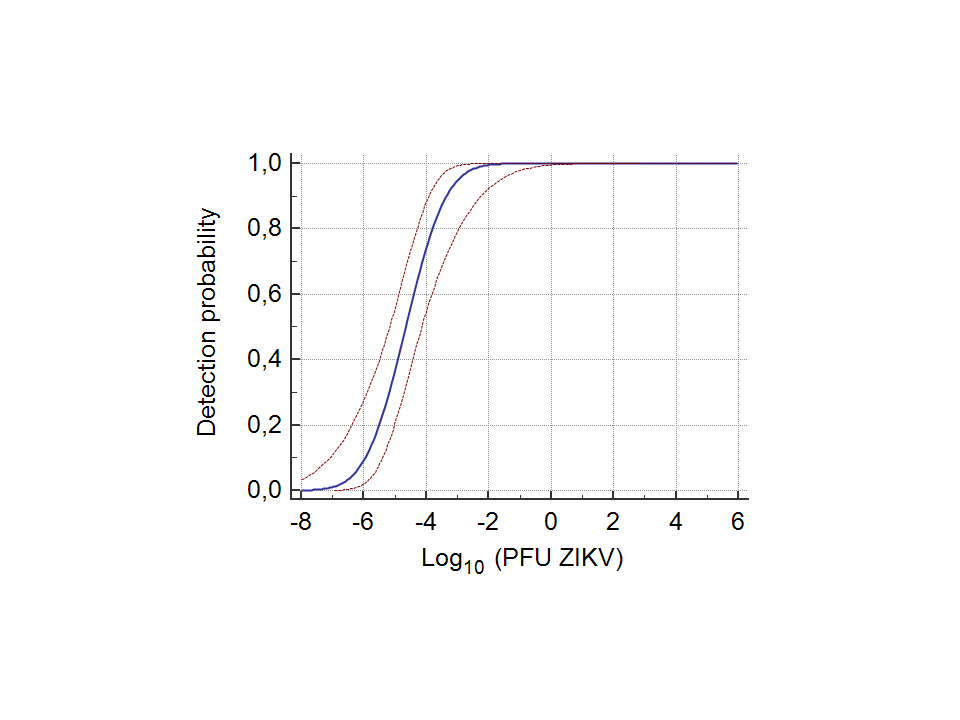
**
